# Surgical considerations in the resection of solitary fibrous tumors of the pleura

**DOI:** 10.1186/s13019-023-02168-7

**Published:** 2023-02-24

**Authors:** Hana Ajouz, Amir Humza Sohail, Hassan Hashmi, Myriam Martinez Aguilar, Sabrina Daoui, Miltiadis Tembelis, Muhammad Aziz, Tirajeh Zohourian, Collin E. M. Brathwaite, Robert J. Cerfolio

**Affiliations:** 1 Department of Surgery, University of Miami Health System, Miami, FL USA; 2 Department of Surgery, New York University Langone Health, New York, USA; 3 Department of Surgery, LeHigh Valley Health Network, Allentown, PA USA; 4 Department of Surgery, Mayo Clinic, Rochester, MN USA; 5 New York University Long Island School of Medicine, Mineola, NY USA; 6 Department of Radiology, New York University Langone Health, New York, USA; 7 Department of Medicine, University of Toledo Medical Center, Toledo, OH USA

**Keywords:** Lung tumor, Solitary fibrous tumor, Surgery, VATS

## Abstract

Solitary fibrous tumors (SFTs) are rare mesenchymal pleural neoplasms with an overall good prognosis and low recurrence rate if completely resected and if degree of differentiation is favorable. Within the last decade, advances in research have led to more reliable methods of differentiating SFTs from other soft tissue tumors. Historically, several markers were used to distinguish SFTs from similar tumors, but these markers had poor specificity. Recent evidence showed *NAB2-STAT6* fusion gene to be a distinct feature of SFTs with 100% specificity and sensitivity. Surgical resection, with an emphasis on obtaining negative margins, is the mainstay of treatment for SFTs. Preoperative planning with detailed imaging is imperative to delineate the extent of disease and vascular supply. One important radiologic distinction to aid delineation of a pleural-based tumor compared to a pulmonary parenchymal-based tumor is the angle that the tumor forms with the chest wall, which is obtuse for a pleural-based tumor, and acute for tumors of the lung parenchyma. Often, preoperative tissue diagnosis is not available, and surgery is both diagnostic and curative. Intraoperatively, emphasis should be on complete resection with negative margins. SFTs are resected via several approaches: thoracotomy, sternotomy with the option of hemi-clamshell extension, video-assisted thoracoscopic surgery, and robotic approach, which is increasingly being used and is our preference. We recommend a minimally invasive approach for most lesions, and have resected SFTs of the pleura that are up to 12 cm with the robotic approach. However, the current literature often cites 5 cm as the cut off for an open thoracotomy. Nevertheless, even with larger tumors, a minimally invasive robotic approach is our preference and practice. For giant SFTs (> 20 cm), an open approach may be preferable. Multiple thoracotomies and rib resection may be required to gain adequate exposure and ensure complete resection in these tumors. However, it is noteworthy that most of these tumors have a soft consistency and thus, once bagged, can easily be removed minimally invasively, and thus minimally invasive approach should not be completely ruled out. Recurrence in SFTs usually results from incomplete resection and redo surgery may portend a favorable prognosis.

## Background

Solitary fibrous tumors (SFTs) were first described, histologically, in 1870 by Wagner, but it was not until 1931 that tumors of the pleura were categorized as either diffuse mesothelioma or localized mesothelioma by Klemperer and Rabin [1]. They have been historically recognized by several names, including benign mesothelioma, pleural fibroma, subserosal fibroma, solitary fibrous mesothelioma, submesothelial fibroma, and localized fibrous tumors [1]. Advancements in microscopy and immunohistochemistry proved instrumental in pinpointing that the origin of this tumor from the submesothelial, noncommitted mesenchymal layer, and not from the mesothelial layer [2]. Therefore, now this entity is referred to as solitary or localized fibrous tumors of the pleura.

It is noteworthy that the data available on SFTs of the pleura, an uncommon neoplasm with an unpredictable prognosis, are limited. SFTs can be classified as benign or malignant. The 2013 WHO classification of soft tissue tumors defines malignant forms as hypercellular, mitotically active (> 4 mitosis/10 high-power fields), with cytological atypia, tumor necrosis, and/or infiltrative margins [3]. There is wide variation in the reported proportion of malignant lesions, with estimates ranging from 30 to 60% [4, 5]. Further, evidence on the clinical presentation, natural history and prognosis of SFTs of the pleura is almost exclusively derived from retrospective case reports and series. Thus, the rarity of SFTs, diversity in histology, combined with changes in diagnostic terminology over time have led to an unsystematic approach to this entity, especially in terms of surgical approach and preoperative planning.

## Epidemiology

Although the exact incidence of SFT is difficult to determine, SFTs constitute a small proportion (< 5%) of all pleural tumors [6]. SFTs may present at any age, but are most commonly seen between the ages of 50–70 years [2]. There does not seem to be a gender predilection with men and women being equally affected [7]. Data have not shown any environmental exposure (such as radiation, tobacco, asbestos, etc.) to be related to SFTs. Further, no hereditary patterns or genetic risk factors have been identified [8].

## Clinical features and presentation

SFTs generally have an indolent onset with slow growth, and no obvious clinical signs or symptoms in the initial stages [8]. Interestingly, most SFTs are discovered incidentally in asymptomatic patients [8]. However, a correlation between the tumor size and symptoms has been observed, especially in tumors > 10 cm in size [9]. Typically, patients present with non-specific pulmonary symptoms, such as cough, shortness of breath, chest pain, hemoptysis, and obstructive pneumonitis [7]. Compressive symptoms are observed in large, especially giant (> 20 cm) SFTs.

Paraneoplastic syndromes, such as Doege–Potter syndrome, Pierre–Marie–Bamberger syndrome, arthralgia, articular edema, and weight loss have also been reported. Of note, refractory hypoglycemia secondary to Doege–Potter syndrome is seen in < 5% of cases [8]. The mechanism of refractory hypoglycemia is related to secretion of insulin-like growth factor 2 (IGF-2) by the tumor [10]. Hypertrophic pulmonary osteoarthropathy (HPO), characterized by clubbing of the fingers, periostitis and synovial effusions, has been reported in up to 22% of patients with SFT [11]. Unfortunately, the mechanism underlying HPO is not entirely clear [11].

## Preoperative evaluation, diagnosis, and surgical planning

Meticulous preoperative planning is critical to safe and adequate resection of SFTs, although a definitive preoperative diagnosis may be difficult to obtain.

### Cardiopulmonary risk evaluation

The initial preoperative risk assessment for surgical resection should begin with a cardiac evaluation to assess for cardiovascular disease, arrhythmias, structural heart disease, and functional status. In patients with cardiac risk, a preoperative electrocardiogram may be indicated to establish baseline electrical function. Following cardiac risk assessment, the forced expiratory volume in one second and the diffusing capacity for carbon monoxide should be measured to assess for pulmonary risk [4].

### Imaging findings and delineation of extent of lesion

**Fig. 1:**
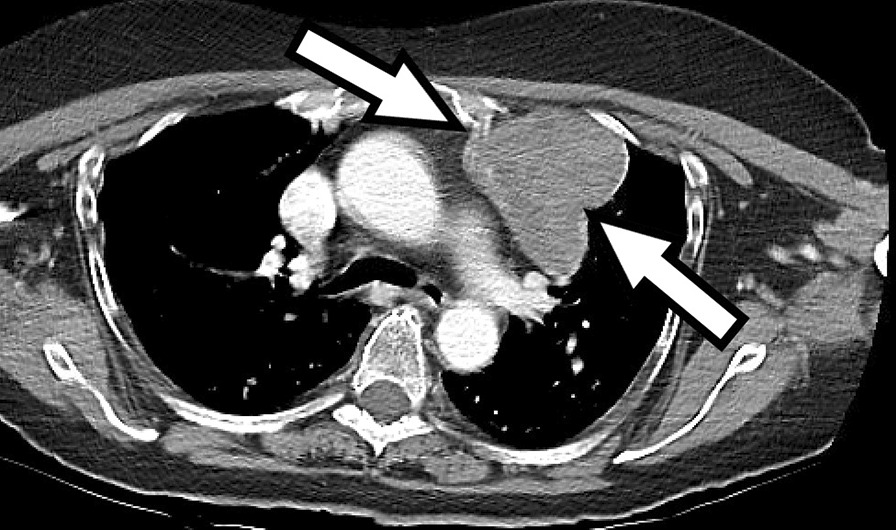
Axial CT image of the chest with IV contrast demonstrates a heterogenous, partially enhancing mass (arrow) along the anterior and mediastinal pleura

SFTs usually appear radiologically similar to other soft tissue tumors with no specific pathognomonic radiological features. SFTs usually present as a homogenous, possibly lobulated, contrast enhancing mass on cross-sectional imaging [8], see Fig. 1.

**Fig. 2:**
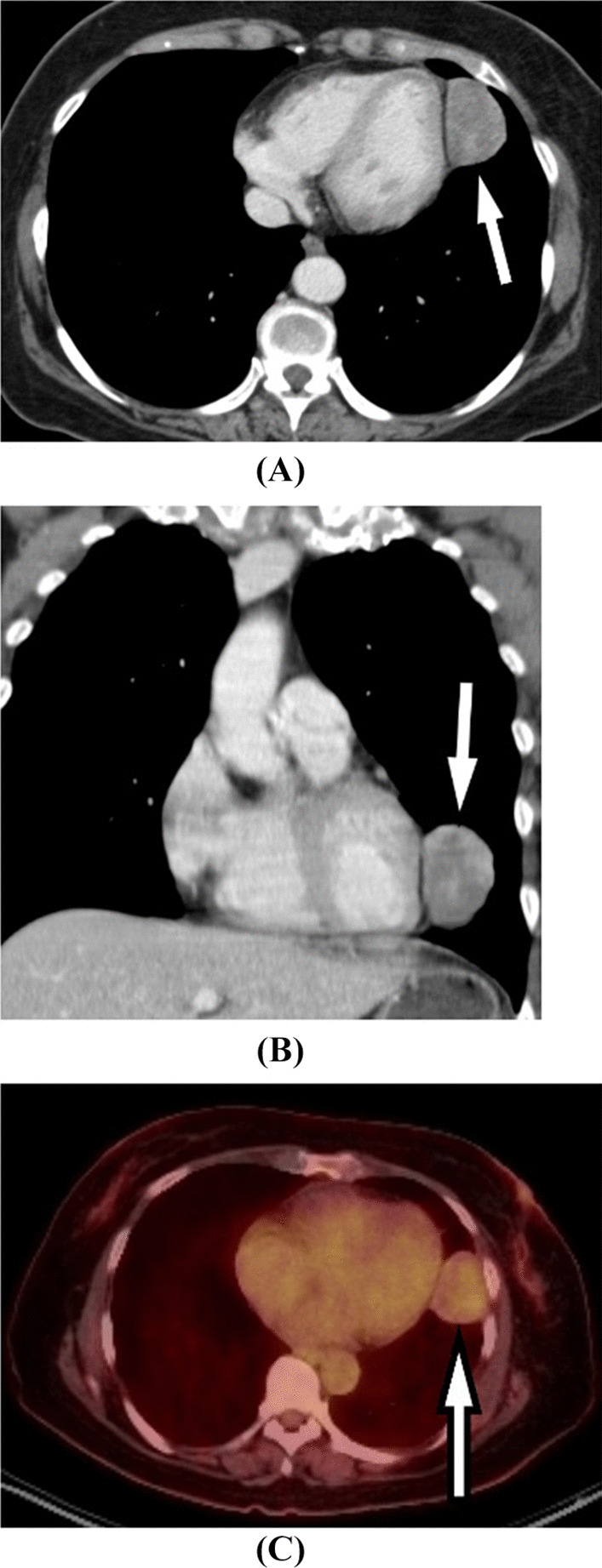
**A** Axial and **B** coronal CT image of the chest with IV contrast demonstrates a heterogenous mass (arrow) forming obtuse angles with the mediastinal pleura. **C** Axial PET-CT image demonstrates the mass (arrow) with moderate FDG uptake

However, cystic areas, calcifications, myxoid degeneration, and hemorrhage may also be present [6]. SFTs tend to arise from the visceral pleura in two-thirds of cases. When it arises from parietal pleura, the mass is often seen as inverted and growing within the lung parenchyma [6]. SFTs tend to displace (less frequently invade) surrounding tissues, making resection more feasible than other more invasive tumors, and rendering prognosis more favorable from an oncologic standpoint. One important radiologic distinction to aid delineation of a pleural based tumor compared to a tumor in the pulmonary parenchyma is the angle the tumor has with the chest wall, which is acute in the case of a tumor in the lung parenchyma and obtuse for a pleural-based tumor, see Fig. 2.

There is wide variation in the reported diameter of SFTs (range from 1 cm to over 40 cm) [12]. SFTs are usually well circumscribed, with a fibrous pseudocapsule [13]. Moreover, they are frequently pedunculated with the pedicle typically containing large feeding vessels. This is especially true in the case of SFTs arising from the visceral pleura (which constitute two-thirds of pleural SFTs), with the lesion being attached to the lung by a narrow pedicle. One-third of pleural SFTs arise from the parietal pleura in which case the mass tends to be larger with a broad-based attachment [14].

A pedicle can be radiologically visualized in 40% of cases [15]. Hence, due to the presence of a pedicle, significant tumor mobility, change in shape and location may be observed in sequential images [2, 13]. Associated pleural effusion can also be seen in some instances [2].

**Fig. 3:**
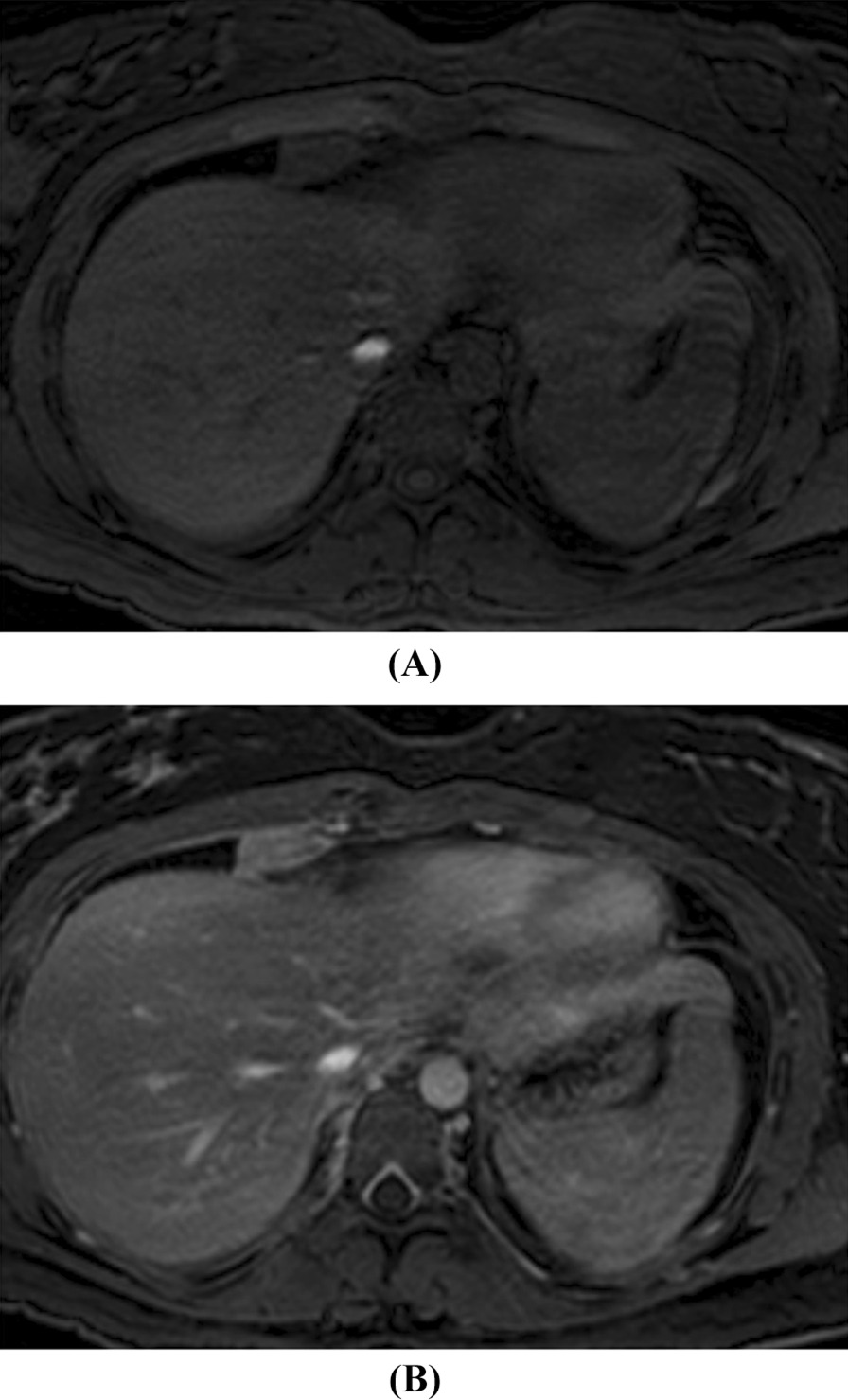
**A** Pre-contrast axial T1-weighted and **B** post-contrast axial T1-weighted MR images of the chest demonstrate a hypointense, avidly enhancing pleural based mass most consistent with a solitary fibrous tumor of the pleura

MRI is of limited value in the assessment of pleural tumors. However, occasionally MRI may be used to assess the relationship and extent of involvement of the mass and adjacent structures, such as mediastinum, the great vessels, vertebrae and the diaphragm [9], see Fig. 3. Imaging is generally of limited value in differentiating benign versus malignant SFTs. Positron emission tomography (PET) scan has minimal utility in diagnosing SFTs because of their low metabolic activity [15]. Furthermore, in case of a non-homogeneous lesion, differentiating it from bronchogenic carcinoma on imaging may pose a diagnostic challenge; this is especially relevant in patients with a history of smoking.

### Vascular supply

Resection of SFTs may prove challenging due to their hypervascular nature and extensive collateral blood supply [16]. Identifying tumor vascularity preoperatively can help prevent massive intraoperative hemorrhage. Therefore, preoperative angiography is a valuable tool for identifying major feeding vessels and surgical planning. The blood supply of SFTs has only rarely been described in literature, but most reports detail aberrant feeding vessels [17]. Guo et al. [18] reported 5 patients with pleural SFTs that had multiple feeding vessels from the internal mammary, bronchial, inferior phrenic, and intercostal arteries. An aberrant blood supply from the celiac trunk has also been reported [19].

### Tissue diagnosis

Preoperative fine needle aspiration (FNA) biopsies are of little diagnostic value since they often contain an inadequate cellular sample, and therefore have poor accuracy (45% accuracy in one series) [4]. On the other hand, core needle biopsies may provide enough material to diagnose SFTs [20]. Unfortunately, the provided sample may still be inadequate to establish evidence of aggressive behavior [13]. Surgical resection is required for accurate histopathologic evaluation of SFTs and is considered the gold standard for diagnosis as well. Given the inadequacy of methods of differentiating benign from malignant lesions, intraoperatively all SFTs should be treated as malignant, especially with the objective of obtaining negative margins to prevent recurrence.

### Preoperative embolization

Preoperative percutaneous embolization has been described in literature and may be useful in lowering intraoperative blood loss as well as alleviating associated symptoms preoperatively, but is rarely needed except for large invasive SFTs or those that are locally aggressive [21]. Furthermore, preoperative embolization may also prove helpful with intraoperative dissection [21]. Andrews et al. reported a case where a patient with a SFT with associated joint pain from hypertrophic pulmonary osteoarthropathy underwent preoperative embolization via the bronchial arterial system, resulting in significant improvement in symptoms [21].

## Surgical considerations

### Surgical approaches

Several surgical approaches have been described in literature for resection of SFTs. Resection can be performed via thoracotomy, sternotomy with the option of hemi-clamshell extension if required, video-assisted thoracoscopic surgery (VATS), or robotic-assisted surgery. We prefer a robotic platform and have used it almost exclusively over the past 7 years. The patient should be positioned in a 45° lateral decubitus position to avoid hemodynamic variations associated with decompressing the heart, the great vessels, and the airway.

Open thoracotomy was generally considered the gold standard of treatment in sessile, large, invasive, and malignant lesions [22]. However, minimally invasive approaches seem promising for the resection of SFTs, although historically the open approach was more commonly used. In a retrospective series, Magdeleinat et al. reported resection of SFTs in 60 patients with an average tumor size of 8.5 cm over 20 years. The approaches employed were 80% posterolateral thoracotomy, 8% anterolateral thoracotomy, 10% VATS and 1% sternotomy [4]. Similarly, Rena et al. reported in their series the surgical approaches as 66% posterolateral thoracotomy, 28% anterolateral thoracotomy and 5% VATS [18].

VATS or a robotic approach can be used for certain suitable tumors, and has several advantages over open approach, including decreased postoperative pain, reduced impairment of respiratory function and more favorable cosmesis. However, conversion to open technique is strongly recommended if margin clearance is not achievable with this approach. Lesions < 5 cm, small pedunculated tumors, and lesions at the periphery of the lung may be more amenable to VATS resection. However, even with larger tumors, if a VATS or robotic resection is deemed technically feasible, it can be attempted. Further, with advances in minimally invasive and robotic technology, and greater surgical expertise, we believe minimally invasive approaches, especially robot-assisted surgery, are likely to become more common. At our institution in New York, our preferred minimally-invasive approach is robotic resection.

For large tumors, a small thoracotomy incision can be added to VATS or robotic approach to remove the specimen safely and completely. It is important to emphasize that utmost care must be taken when removing these tumors from the chest cavity, since contact metastases have been reported [23]. It is especially important to note that when a minimally invasive approach is employed, the port sites must be protected since port site seeding and recurrence have been reported with SFTs [24].

Amore et al. reported the first case in literature of an SFT in anterior mediastinum undergoing complete surgical resection by robotic-assisted approach. In this case, the resected mass was well-circumscribed in nature, with no invasion of vital neurovascular structures and measured 10 cm × 6 cm × 8 cm in size [25]. The surgical approach in this case involved a three-port access in the left sixth intercostal space in the anterior axillary line, the left third intercostal space in the anterior axillary line and at the left sixth intercostal space in the midclavicular line. The authors reported the use of a cautery spatula and a Cadiere clamp for dissection and handling of the tumor.

### Other intraoperative considerations

Surgical management of SFTs is similar to soft tissue sarcomas with the primary goal of wide resection with microscopically negative margins and preservation of any critical surrounding structures. Surgical planning and intervention is highly variable given the variable location of the primary tumor and involved structures. As mentioned above, SFTs are often difficult to resect completely due to their hypervascular nature and extensive collateral vessels. Massive life-threatening hemorrhage can occur due to their hypervascular nature, so it is important to identify the vascular pedicle and ligate it first to limit any bleeding complications [26]. Of note, extensive adhesions around SFTs may lead to difficulty in identification and ligation of feeding and hilar vessels, particularly in case of a lesion with a broad-based pedicle and a limited and suboptimal operative window [23]. All of these factors contribute to an overall reported operative mortality between 1.5 and 12% [22].

While complete surgical resection with a wide margin is recommended, the extent of resection is usually decided on a case-by-case basis upon detailed appraisal of the situation intraoperatively. An isolated mass excision may be achievable in certain instances whereas an *en bloc* resection of surrounding structures is required in most of the remaining cases [4, 13, 27]. The main determinants of the extent of resection are the identification of invasion of surrounding structures and complete resection with negative margins. En bloc resection of diaphragm, parietal pleura and pericardium may be required to achieve complete resection.

The distinction between SFTs arising from visceral and parietal pleura is of particular importance. Tumors arising from the parietal pleura are more difficult to resect since in general they tend to be large and affixed to the chest wall [17]. SFTs arising from the visceral pleura can often be resected with minimal impact on adjacent structures, whereas those arising from the parietal pleura may require chest wall resection to obtain a negative margin. Surgical planning is further complicated by the fact that often it is difficult to ascertain preoperatively if the tumor arises from the visceral or the parietal pleura. When the tumor involves the lung, a wedge resection or a lobectomy may be needed to obtain a clear margin.

The wholeness of surgical excision is a major prognostic factor for both benign and malignant SFTs [9, 15]. Obtaining adequate negative microscopic margins has been shown to decrease the rate of local disease recurrence and possibly improve survival. It is pertinent to mention that peritumoral adhesions are common (60% in one series) [4]. To obtain negative margins, all peritumoral inflammatory adhesions should be resected since they might be positive for tumor microscopically and thus lead to recurrence [27]. Several authors have recommended a liberal use of intraoperative frozen section histopathology to ensure negative margins [4].

It is noteworthy that SFTs of the pleura do not generally have lymphatic spread, and therefore a formal lymphadenectomy is not required, especially if the diagnosis of SFT has been established preoperatively.

### Resection of giant solitary fibrous tumors

Giant solitary fibrous tumor (> 20 cm) of the pleura is a rare tumor that has been only occasionally reported in scientific literature, and usually as single cases [25, 28, 29]. Like all SFTs, the gold standard of treatment for giant SFTs is also complete resection. However, this is usually challenging in case of giant SFTs.

Large tumors with an invasive behavior may be difficult to resect through a standard posterolateral thoracotomy. There is no standard approach for such cases and in reported cases incisions were tailored to the size of the tumor and its exact location, see Tables 1 and 2. For instance, Yanagiya et al. described an extended thoracotomy combined with a subcostal incision as a useful approach for surgical removal of giant SFTs compressing the diaphragm [30]. Furukawa et al. reported having performed the surgery via two thoracotomies at two different levels [31]. Their initial thoracotomy site was at the fifth intercostal space, which was followed by another incision at the eighth intercostal space. Filosso et al. used a right posterolateral thoracotomy with resection of the fifth, sixth, and seventh rib to resect a 20 cm × 20 cm × 12 cm tumor originating from the parietal pleura, without any pedicle [32]. Song et al. did a two stage surgical treatment of a 27 cm × 11 cm × 12 cm tumor [12]. First, an anterior thoracotomy was performed on the third intercostal space at the sternal margin to divide the feeding vessel to the tumor, then the tumor was exposed through lateral thoracotomy at the fifth intercostal space. The mass was not invading lung tissue and therefore no lobectomy or wedge resection was performed.

**Table 1:** Literature review of different surgical approaches used for resection of SFTs

References	Surgical approach
Cardillo et al. [13]	71% video-assisted thoracic surgery, n = 39
	18% posterolateral thoracotomy, n = 10
	7% lung resection, n = 4
	2% thymectomy, n = 1
	2% *en bloc* chest wall resection, n = 1
	Total cases = 55
Lococo et al. [27]	70% *en bloc* resection, n = 35
	26% isolated mass excision, n = 13
	4% no surgical intervention performed, n = 2
	Total cases = 50
Magdeleinat et al. [4]	80% posterolateral thoracotomy, n = 48
	10% video-assisted thoracic surgery (VATS), n = 6
	8% anterolateral thoracotomy, n = 5
	2% median sternotomy, n = 1
	Total cases = 60
Suter et al. [5]	100% thoracotomy, unspecified, n = 15
	Total cases = 15

**Table 2:** Comparison of different surgical approaches used for giant SFTs

Author	Surgical approach	
Yanagiya et al. [30]	Extended thoracotomy combined with subcostal incision	
	6th rib was transected	
	Tumor was resected en bloc	
	No lobectomies or wedge resection	
	Pathology: 18 cm SFT	
Filosso et al. [32]	Right posterolateral thoracotomy	
	5th, 6th, and 7th ribs resected	
	No lobectomies; large wedge resection	
	Pathology: 20 × 20 × 12 cm Malignant sessile SFT originating from parietal pleura	
Song et al. [12]	Two stage surgical treatment	
	Anterior thoracotomy was performed on the third intercostal space to ligate feeding vessels	
	Tumor was exposed through lateral thoracotomy at the 5th intercostal space	
	No lobectomy or wedge resection	
	Pathology: 27 × 11 × 12 cm SFT	
Furukawa et al. [31]	Left posterolateral thoracotomy through the fifth and eighth intercostal spaces	
	Fifth intercostal space as initial Thoracotomy site	
	Then incision through the eighth intercostal space	
	Wedge resection	
	Pathology: 20 × 19 × 15 cm SFT	

However, it is noteworthy that most of these tumors have a soft consistency and thus once bagged are easily able to be removed minimally invasively, and thus minimally invasive approach, although less likely to be effective, should not be completely ruled out, especially if anatomy and location are favorable.

### Recurrence and redo surgery

There is no evidence of correlation between karyotype, histology, anatomic location and clinical outcomes. More specifically, histologic examination failed to predict malignant behavior, because SFTs are composed of several tumor cell populations with various degrees of differentiation. There are several hypotheses about different molecular or genetic components that might be of prognostic value. However, none are ready for clinical use, either alone or in conjunction with the current scores that are based on clinicopathologic features [20, 23, 33, 34]. In addition, the utility of the staging system of the American Joint Committee on Cancer to predict prognosis for SFTs is unclear.

Although the majority of SFTs behave in an indolent fashion, a small proportion are aggressive and may recur despite adequate resection (10–25% of tumors recur by 10 years post-operatively) [35]. In most series, recurrence of SFTs was found to be due to incomplete surgical resection, metastatic disease at presentation, tumor size > 10 cm, a high mitotic rate, presence of tumor necrosis or tumor seeding within the adhesions [9, 13, 15, 20, 32, 33, 35]. Of note, when a VATS approach is employed, the risk of port site seeding and the resulting recurrence is also a concern. Late relapse is common even in tumors with benign histological features. Relapse has been reported up to 20 years after initial presentation [35]. This highlights the importance of continued long-term follow-up even for tumors perceived to be benign. We recommend a CT scan every 6 months for the first 2 years following surgical resection, then a yearly CT scan from then on. If the lesion was PET positive preoperatively, a PET scan may be included in postoperative surveillance.

Recurrence is generally located in the resection area [35]. Re-resection is indicated in the absence of disseminated disease [28]. Thus, an aggressive surgical approach should be employed in cases of recurrence if the lesion is surgically resectable [4]. Prolonged survival after re-resection of a recurrent SFT recurrence is possible [15, 35]. Chemotherapy plays a minor part in treatment of SFTs, and is generally indicated as salvage or palliative therapy, although neoadjuvant chemotherapy has been reported with mixed response at best [21].

## Conclusion

Early resection of SFTs with a focus on obtaining negative margins is the standard of care and is also diagnostic in certain cases. Preoperative imaging and angiography is vital in surgical planning. Surgical approach must be tailored to location of tumor, extent of disease and involvement of surrounding structures. Certain lesions may be amenable to minimally invasive approaches, such as VATS and robot-assisted surgery. Careful long-term follow up is needed for the early detection of growth of the residual tumors or recurrence. In case of recurrence, re-do surgery may offer the best chance of long-term survival.

## Data Availability

Not applicable.

## Abbreviations

CT: Computed tomography
SFTs: Solitary fibrous tumors
VATS: Video-assisted thoracoscopic surgery
IGF-2: Insulin-like growth factor 2
